# A toxin-antidote CRISPR gene drive system for regional population modification

**DOI:** 10.1038/s41467-020-14960-3

**Published:** 2020-02-27

**Authors:** Jackson Champer, Esther Lee, Emily Yang, Chen Liu, Andrew G. Clark, Philipp W. Messer

**Affiliations:** 1 Department of Computational Biology, Cornell University, Ithaca, NY 14853 USA; 2 Department of Molecular Biology and Genetics, Cornell University, Ithaca, NY 14853 USA

**Keywords:** Genetic engineering, Protein design, Population genetics, CRISPR-Cas9 genome editing

## Abstract

Engineered gene drives based on a homing mechanism could rapidly spread genetic alterations through a population. However, such drives face a major obstacle in the form of resistance against the drive. In addition, they are expected to be highly invasive. Here, we introduce the Toxin-Antidote Recessive Embryo (TARE) drive. It functions by disrupting a target gene, forming recessive lethal alleles, while rescuing drive-carrying individuals with a recoded version of the target. Modeling shows that such drives will have threshold-dependent invasion dynamics, spreading only when introduced above a fitness-dependent frequency. We demonstrate a TARE drive in *Drosophila* with 88-95% transmission by female heterozygotes. This drive was able to spread through a large cage population in just six generations following introduction at 24% frequency without any apparent evolution of resistance. Our results suggest that TARE drives constitute promising candidates for the development of effective, flexible, and regionally confinable drives for population modification.

## Abstract (web summary)

CRISPR homing gene drives are highly invasive and can fail due to the rapid evolution of resistance. Here the authors present TARE drive, inspired by naturally occurring selfish genetic elements, which is less vulnerable to resistance and can potentially be confined to a target population.

## Introduction

Gene drives have the potential to rapidly spread through a population by biasing inheritance in their favor^1–7^. These systems could be used for population modification by carrying a payload allele engineered for a particular purpose, such as a transgene that reduces the capacity for malaria transmission in mosquitoes^1–3,5^. Gene drives may also be used for the direct suppression of a population, for example by targeting an essential but haplosufficient gene. Such suppression-type drives have been touted as potential control strategies for disease vectors, invasive species, or agricultural pests^1–3,5^.

CRISPR-based homing drives promise a flexible gene drive mechanism for both population modification and suppression, and such systems have now been demonstrated in a variety of organisms, including yeast^8–11^, flies^12–18^, mosquitoes^19–21^, and mice^22^. These constructs work by cleaving a wild-type allele at a predetermined target site. The drive allele is then copied into the cleaved site during homology-directed repair, converting heterozygotes for the drive allele into homozygotes in their germline. However, the spread of CRISPR homing drives is typically thwarted by the formation of resistance alleles when Cas9 cleavage is repaired by end-joining, which tends to generate indel mutations at the target site^12,13,15–18,20–23^. This can take place both in the germline as an alternative to drive conversion and at the early embryo stage due to cleavage by maternally deposited Cas9^15^. While several strategies for reducing the rate of resistance allele formation have already been successfully tested, including gRNA multiplexing^16^ and improved promoters^16,23^, these improvements were not sufficient to reduce resistance rates to an acceptably low level.

Recently, a CRISPR-based population suppression drive that combined an improved promoter with a carefully selected target site where resistance alleles are nonviable was shown to be capable of suppressing small cage populations of *Anopheles gambiae*^24^. While promising, such a strategy may not be easily adoptable for approaches where the aim is population modification rather than suppression, and computational modeling has indicated that even high-performance population suppression systems may still face substantial evolutionary and ecological obstacles^25^. CRISPR homing gene drives also require Cas9 cleavage in the germline during a narrow temporal window in order to facilitate homology-directed repair instead of end-joining, as the latter will typically lead to the formation of resistance alleles. This increases development difficulty when designing homing drives in new species due to the need for a suitable promoter. Thus, flexible population modification systems that minimize formation of resistance alleles may be needed for use either alone or in combination with a population suppression system.

One possible strategy for reducing resistance allele formation is to remove the need for homology-directed repair altogether. This criterion is fulfilled by drives that use the “toxin-antidote” principle, a strategy employed by many naturally occurring selfish genetic elements^26^. Indeed, a toxin-antidote system in *Drosophila melanogaster*^27^, based on the *Medea* system found in flour beetles^28^, was one of the first engineered gene drive systems to successfully spread through an experimental cage population. However, because it uses elements that are highly specific to *Drosophila*, such a system has proven difficult to engineer in other species. Other designs for engineered toxin-antidote systems also exist^29–34^, but critical elements for their construction would likely be difficult to identify. One possibility for how such a system could be constructed in a more flexible manner is by engineering a drive allele that contains Cas9 and gRNAs to serve as the “toxin” by targeting a haplosufficient gene where disrupted alleles are recessive lethal. The drive allele would also contain an “antidote”, consisting of a recoded copy of the target gene that cannot be targeted by the gRNA. Such a drive would be expected to steadily convert wild-type target alleles to disrupted alleles, at which point they would be removed from the population in embryos where no drive or wild-type allele is present to provide rescue. We term such a system TARE (toxin-antidote recessive embryo) drive.

In addition to minimizing resistance, a TARE drive would also be expected to exhibit threshold-dependent invasion dynamics. This can constitute a solution to one of the key problems of homing-type gene drives: the ability of such drives to invade any population connected to the release population by low levels of gene flow^35^. Such highly invasive “global” drives would be problematic whenever a drive is required to be confined to a specific population, such as an island or a continent^36^. By contrast, TARE drives with an invasion threshold could remain confined to contiguous populations without being able to invade sufficiently distant populations through occasional migrants. By this means, they may also provide a critical component in enabling so-called “tethered” drives, which could be used for both population modification and suppression strategies^37^.

A recent study has provided the first demonstration of a “distant-site” TARE drive, termed ClvR, where the drive allele and target gene reside at two different genomic loci^38^. ClvR was able to successfully spread through small population cages^38^. Here, we demonstrate a “same-site” TARE system, where the drive allele and target gene are at the same locus. Such a system may have several advantages over the distant-site system, particularly since the rescue element uses the target gene’s natural promoter elements, potentially increasing the chance of efficient rescue. We show that our TARE system successfully biases inheritance of the drive allele and reaches all individuals in a large *Drosophila* population cage after just six generations following a modest size release, without any apparent formation of resistance alleles.

## Results

### TARE drive mechanism and design considerations

**Fig. 1:**
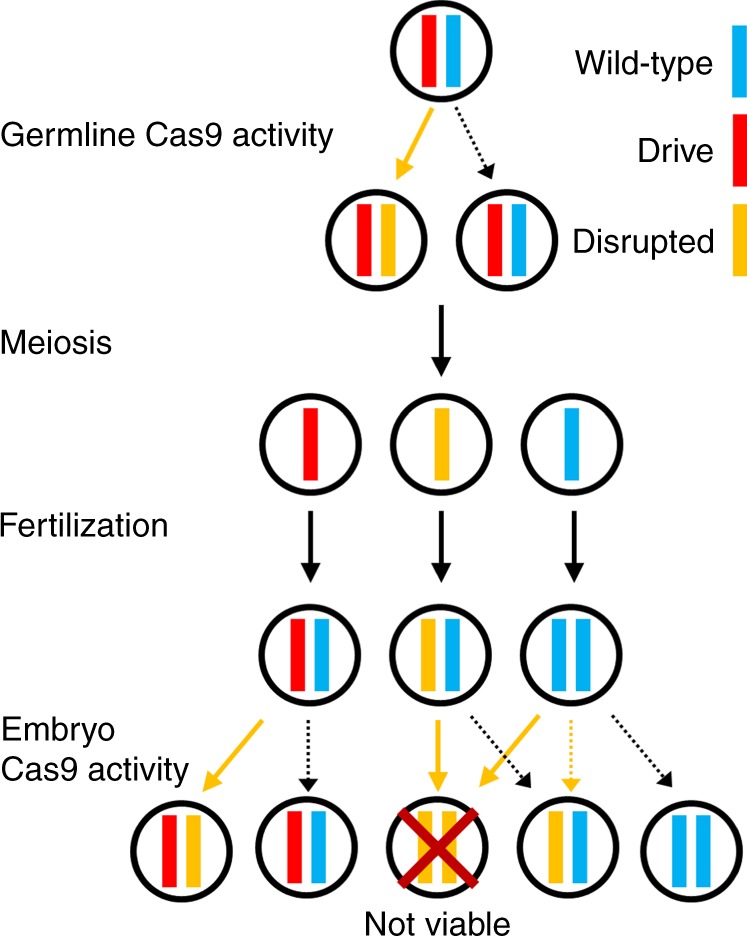
Mechanism of the TARE drive. In the germline of drive/wild-type heterozygotes, wild-type copies will usually undergo cleavage followed by homology-directed repair or end-joining, either of which will result in a disrupted target gene and loss of function. Meiosis and fertilization (shown here by a wild-type gamete) then occur. In the progeny of females with the drive, maternally deposited Cas9 and gRNA will cleave most wild-type alleles, which will become disrupted after end-joining repair. Any individual that inherited two recessive lethal disrupted alleles of the target gene will be nonviable, which will lead to a systematic increase of the relative frequency of the drive allele over time. Dotted arrows specify events that should occur less frequently.

The “same-site” TARE drive consists of a drive element placed inside a haplosufficient gene where disrupted alleles are recessive lethal. The presence of the drive disrupts the wild-type version of the gene, yet the drive construct contains a recoded version of a portion of this gene sufficient to restore its function, as well as a set of gRNAs that target only the wild-type gene (but not the recoded version) at one or more target sites. Cleavage of the target gene by the drive creates a disrupted allele (typically termed “r2 resistance allele” in studies on homing drives, which are distinguished from the “r1” resistance alleles that maintain gene function). Individuals that possess two such disrupted alleles will be nonviable, removing such alleles from the population (Fig. 1). As a result, the relative frequency of the drive allele over the wild-type allele will increase over time.

When cleavage of the target gene occurs in the germline, this can create a disrupted gene either through end-joining repair or by homology-directed repair around the cut site using the right target fragment in the drive allele, converting the wild-type allele to the disrupted sequence form used for the template. In the progeny of females with the drive, maternally deposited Cas9 and gRNA will also result in cleavage of wild-type alleles in the embryo, creating disrupted alleles by end-joining and perhaps occasionally homology-directed repair^24^.

The recoded portion of the drive must be designed such that it cannot be targeted by the drive’s gRNAs, nor have sequence homology around these cut sites. Furthermore, the target site of the TARE drive needs to be sufficiently different from the one at which the drive is introduced in order to prevent the entire drive from being copied by homology-directed repair after cleavage. This also helps prevent homology-directed repair from copying only the recoded region and forming an r1 allele. The chance of this can be further reduced if the target site is placed far from the target’s 3′UTR (if the native form is used in the drive’s rescue element—use of another 3′UTR should prevent the need for this).

Limiting the rate at which undesired homology-directed repair events occur is critical for the design of an effective TARE drive, as they could lead to the formation of r1 resistance alleles if repair results in copying of the recoded region, but not the payload gene. However, previous studies have shown that the efficiency of homology-directed repair in homing drives already decreases by ~15% when the distance between cut sites and homology templates is ~100 nucleotides on just one side^39^. For larger distances of 1000 nucleotides or more from cuts sites to repair template, drive efficiency in a multiple-gRNA homing drive fell over 50%^12^ compared to similar drives with no distance between cut sites and templates^15–17^. It is also possible that most or all of these remaining instances of homology-directed repair resulted from the fraction of events where the outer gRNAs of the drive cleaved simultaneously, thereby creating a scenario with effectively no distance between the cut sites and repair template on either side. Thus, we believe that placing cut sites and homology templates over several hundred nucleotides apart from each other should reduce the rate of undesired homology-directed repair considerably. This is particularly the case in TARE drives because the region around the cut sites will also have homology to the remaining fragment of original target gene DNA downstream of the drive allele, which will serve as a superior template for homology-directed repair compared to more distant elements on the opposite side of the drive.

### TARE drive population dynamics

**Fig. 2:**
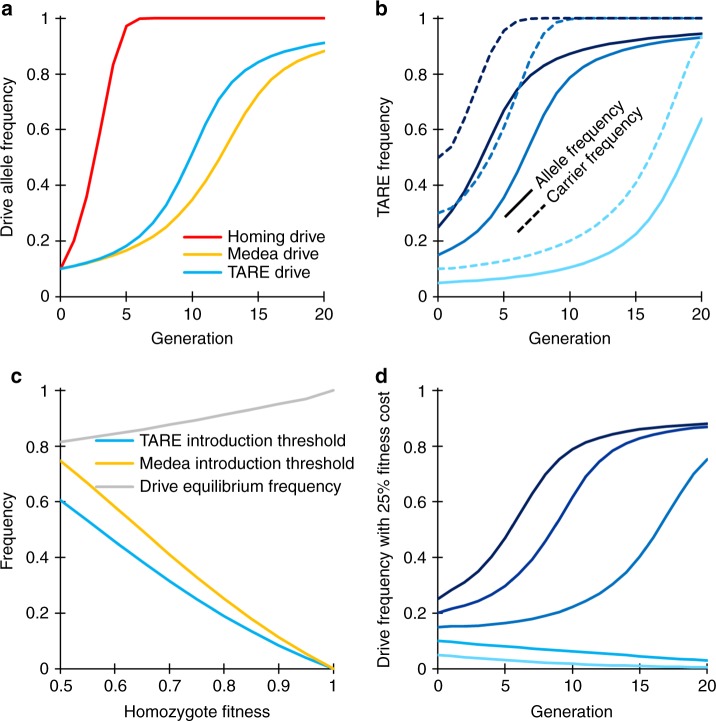
TARE drive dynamics. Expected drive trajectories for ideal drives were simulated in a deterministic model of a single panmictic population with an initial release of drive/wild-type heterozygotes and assuming no fitness costs. **a** An ideal TARE drive increases in frequency less rapidly than an ideal homing drive. It has similar dynamics to an ideal *Medea* drive, but with slightly increased speed to fixation since both male and female drive individuals contribute to the disruption and subsequent removal of wild-type alleles. **b** A TARE drive is expected to show frequency-dependent dynamics, increasing in frequency more rapidly at moderate frequencies than at low frequencies. At high frequencies, however, the rate at which wild-type individuals are removed is slowed. Nevertheless, the drive should rapidly reach all individuals in a population (in the sense that most individuals should carry at least one copy of the drive) with a moderate initial release size. **c** Invasion threshold frequencies of drive heterozygotes as a function of the fitness cost of the drive in homozygotes. These thresholds represent unstable equilibria above which the drive increases in frequency and below which the drive is removed. With fitness costs, both TARE and *Medea* drives will not reach fixation, but instead reach an equilibrium frequency as shown, which is the same for both types of drives. Note that all individuals at equilibrium still have at least one copy of the drive allele. **d** Drive allele frequency dynamics when assuming a drive homozygote fitness equal to 75% that of wild-type individuals, which yields a threshold heterozygote release frequency of 25% (12.5% allele introduction frequency).

We performed simulations of a TARE drive to explore the expected population dynamics of such a system and study how it would compare with other types of drives. Our model suggests that a TARE drive will generally spread more slowly than a homing drive and instead have dynamics similar to a *Medea* system^27,40–42^, although spreading somewhat more quickly (Fig. 2a). All individuals rapidly become drive carriers with at least one copy of the TARE drive, particularly at higher release frequencies, but it can take quite long for the drive to eventually reach its maximum allele frequency (Fig. 2b).

One interesting feature of a TARE drive system is that it should exhibit threshold-dependent dynamics when the drive allele imposes any fitness cost on the organism that goes beyond the reduction in offspring number due to nonviable embryos as a result of the drive mechanism (Fig. 2c). Such an additional fitness cost could be due to a payload allele, result from intrinsic drive activity such as expression of the endonuclease, or be caused by the presence of certain drive elements such as enhancers at a particular genomic locus. When introduced above its characteristic frequency threshold, a drive allele is expected to further increase in frequency, whereas it is expected to decrease in frequency and ultimately be lost when introduced below this frequency (Fig. 2d), similar to the *Medea* system^27,40–42^ (note that in a realistic population of finite size, stochastic effects such as genetic drift could potentially push the drive frequency above or below this threshold). Such threshold-dependent dynamics could be desirable for enabling drives to be confined to certain regions, since it would prevent establishment in other regions through a small number of migrating individuals. This is in stark contrast to homing-type drives, which are self-sustaining at any introduction frequency in deterministic models.

It should be noted that Fig. 2c shows introduction thresholds, referring to the frequency above which a drive will spread following a single release. This is a suitable parameter to consider in assessing invasiveness for initial releases, as well as scenarios where migration events to a nontarget population are rare and typically consist of just a few individuals (which are then quickly removed by selection because of the fitness costs of the drive). However, in models of two demes connected by constant migration, a migration rate threshold will be more informative in determining whether a drive that is fixed in one deme can successfully invade the other deme. Such migration rate thresholds based on continual influx of drive alleles will be significantly lower than introduction thresholds based on a single influx of drive individuals. This is because migrant frequencies are expected to accumulate over time in a scenario of continuous migration (even in the presence of selection acting against the drive), as seen in models of underdominance two-locus^43–46^ and one-locus^46–48^ systems, as well as *Medea*^46^. However, the existence of one threshold generally implies the existence of the other.

Analogous to *Medea*^27,40–42^, a TARE drive with any additional fitness costs will not typically go to allele fixation in the population but will reach an equilibrium frequency between drive alleles and disrupted alleles (Fig. 2c). This is because drive-carrying homozygotes have somewhat lower fitness than drive/wild-type heterozygotes, which is balanced by loss of some offspring without drive alleles when heterozygotes mate with each other. Unless fitness costs are severe, this equilibrium frequency will be quite high, and all individuals will still possess at least one copy of the drive allele (Fig. 2b). This should render TARE drives quite effective for most population modification strategies.

### Drive construct design

**Fig. 3:**
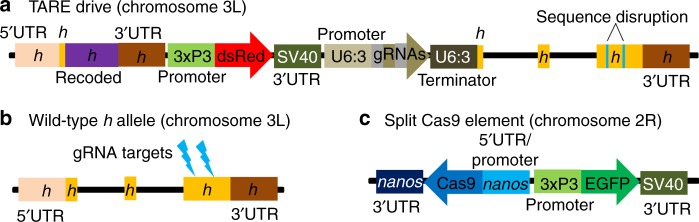
Schematic of the TARE split-drive constructs. **a** The TARE drive is inserted into the coding region of the first exon in *h*. The drive element contains a recoded version of *h* and its 3′UTR, a dsRed marker gene expressed by a 3xP3 promoter together with an SV40 3′UTR, and a gRNA gene consisting of two gRNAs that target *h*, linked by tRNAs and expressed by the U6:3 promoter. **b** The wild-type *h* allele is targeted in the coding sequencing of the third exons by the two gRNAs. **c** The supporting element contains Cas9 expressed by the *nanos* promoter with a *nanos* 3′UTR, and an EGFP marker gene expressed by a 3xP3 promoter together with an SV40 3′UTR.

We designed a TARE drive at the *h* locus in *D. melanogaster*. Our construct consists of a recoded *h* sequence followed by its natural 3′UTR, a dsRed payload gene expressed by the 3xP3 promoter for expression in the eyes (for phenotyping in our *w*^*1118*^ line), and a set of two gRNAs expressed by the U6:3 promoter (Fig. 3a). Our recoded region of *h* starts partway into the first exon and changes each codon, when possible to the most commonly used one for each amino acid in *D. melanogaster*, or the second most common if the most common is already in use (Supplementary Fig. 1). The exception to this is if recoding would result in six repeated nucleotides in a row, in which case the next most common codon was used, or the original codon if no other codons were available. Both introns were also eliminated from the recoded sequence. The gRNA gene contains a tRNA at the start and another in between the gRNAs. The tRNAs are spliced out from the transcript, resulting in mature gRNA sequences. The purpose of this is to allow both gRNAs to be expressed in a single transcript by one promoter, reducing the overall size of the drive system and limiting the need to find multiple suitable promoters (or repeating a promoter sequence, which may cause genomic instability^12^).

The gRNAs of the drive target a region of *h* located approximately 1,400 nucleotides downstream from the drive allele (Fig. 3b). This design should limit the rate of homology-directed repair copying of the whole drive allele (Supplementary Fig. 2), because if both target sites are cleaved, the left end and right ends will only have immediate homology to the disrupted exon downstream of the TARE drive allele, which should serve as the preferred template for homology-directed repair. On the left end of the cut sites, homologous sequences will only be present on the left end of *h*, almost 1400 nucleotides away. It would be even more difficult for only the rescue element to be successfully copied by homology-directed repair. This is because in addition to the large distance to a homologous template on the left side, homologous sequences are also over 350 nucleotides away from the right cut site to the 3′UTR where the sequence matches the drive (Supplementary Fig. 2). Since end resection averages several hundred nucleotides^49^, the template on the left site should only occasionally have the possibility of being used for homology-directed repair, and on both sides, the disrupted target gene fragment (Supplementary Fig. 3) will be preferred due to immediate homology on either side of the cut sites (Supplementary Fig. 2). Furthermore, if incomplete homology-directed repair of the recoded region occurs, it would be unlikely to copy the entire large recoded element, and such events would therefore not be expected to form a resistance allele in most instances.

Because *h* is a haplosufficient gene where disrupted versions are recessive lethal, embryos must have at least one functional copy to survive. This can be a wild-type allele, a drive allele, or an r1 resistance allele in which the *h* gene remains functional despite a change in sequence at both target sites (although we did not detect such r1 alleles in this study). Any embryo receiving two copies of *h* that have both been disrupted by Cas9/gRNA cleavage will be nonviable. The split Cas9 element expressed by the germline *nanos* promoter and containing an EGFP reporter was constructed in a previous study (Fig. 3c)^17^. This Cas9 allele was located on chromosome 2R, while the drive allele in *h* was located on chromosome 3L, so both alleles are expected to segregate independently.

### Drive evaluation

**Fig. 4:**
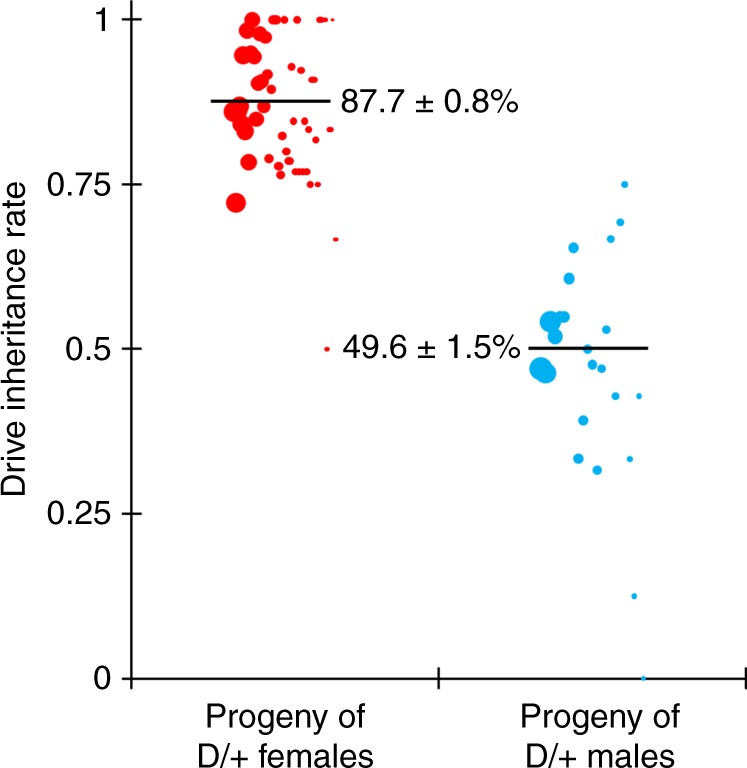
Drive allele inheritance. Females and males heterozygous for the drive allele and the Cas9 allele were crossed with *w*^*1118*^ individuals, and their progeny were phenotyped for dsRed, which indicates the presence of the drive. Females showed biased inheritance of the drive, since many individuals without the drive had two disrupted copies of *h* and thus were nonviable. Half of the progeny of male drive heterozygotes received the drive, since individuals that received a disrupted copy of *h* still received a functional wild-type copy from their mother that remained undisrupted in the absence of maternal Cas9 activity. The size of the dots represents the sample size of adult progeny from a single drive individual. The rate estimates and corresponding estimates of the standard error of the mean (SEM) are displayed, obtained from pooling offspring from all crosses of the same type together (*n* = 1598 for the progeny of females and *n* = 706 for the progeny of males). An alternative statistical analysis that accounts for potential batch effects was also performed but yielded overall similar rates with only slightly increased error estimates (Supplementary Methods, Source Data: Data Sets 1−2).

To assess drive efficiency, we crossed males homozygous for the TARE drive allele to females homozygous for the *nanos*-Cas9 allele. The progeny of these were heterozygous for both the drive and the Cas9 allele. They were then each crossed to *w*^*1118*^ flies, and the progeny were phenotyped. We found that the progeny of heterozygote females was 87.7% dsRed (Fig. 4, Supplementary Data Set 1), which represented a significant deviation from Mendelian inheritance (*p* < 0.001, Fisher’s exact test). Note that this value was obtained from an analysis where we pooled the offspring from all vials together. Such an approach could be confounded by batch effects between groups of progeny that were reared in different vials from different parents. To account for the possibility of batch effects, we also used a generalized linear mixed-effect model to fit our data (see Supplementary Methods). This analysis yielded similar rate estimates with somewhat increased errors (Supplementary Data Sets 1−3).

The high drive inheritance rate we measured for our drive was likely due to lower viability among flies that did not inherit a drive allele. Nearly all wild-type *h* alleles of these flies were likely disrupted in the germline, and a high proportion of paternal *h* alleles were then disrupted by maternal Cas9 activity, resulting in the death of the embryos where cleavage took place. Embryos that inherited the drive allele would remain viable, regardless of maternal Cas9 activity. These results are further supported by subsequent sequencing of the target locus and analysis of the resulting sequences (Supplementary Table 1). We detected wild-type sequences in one out of six flies that inherited the drive allele, but all six sequenced flies that did not receive the drive allele had a clear wild-type sequence. The progeny of males that were heterozygous for the drive allele and the Cas9 allele did not show altered inheritance (Fig. 4, Supplementary Data Set 2). Sequencing revealed that only one out of six such flies that did not inherit the drive had a fully wild-type target sequence, which supports the notion that most wild-type alleles were cleaved in the germline.

Flies inheriting the drive from the above cross were most likely heterozygous with a drive and a disrupted *h* allele. To distinguish germline and maternal Cas9 activity, such heterozygous females that also inherited the Cas9 allele were crossed to *w*^*1118*^ males, and the progeny were then scored as above. In this cross, dsRed inheritance was 95.1%, which was significantly higher than for drive/wild-type heterozygotes (*p* < 0.001, Fisher’s exact test). This implies that germline cleavage and disruption is somewhat less effective than 100% for this drive, because the rate of cleavage in the early embryo was likely the same for both crosses.

**Fig. 5:**
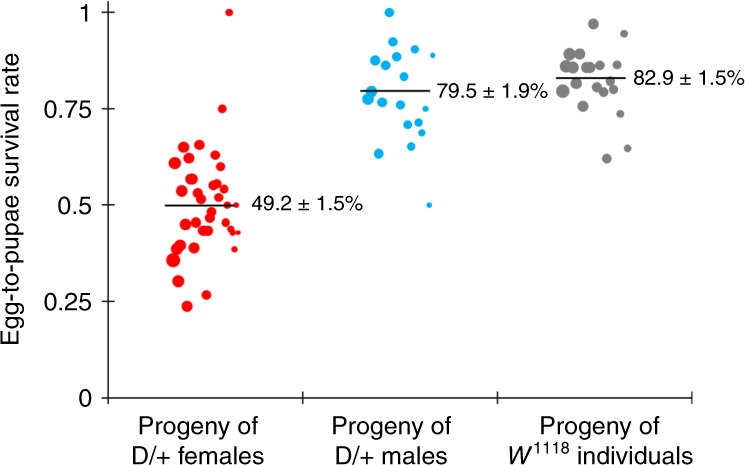
Egg-to-pupae viability. Females and males heterozygous for the drive allele and the Cas9 allele were crossed with *w*^*1118*^ individuals, and *w*^*1118*^ males and females were crossed together. Eggs were counted in 20-h intervals, and pupae were counted 9 days later. Eggs from female drive heterozygotes had substantially lower viability, as expected with a mechanism in which maternal Cas9 activity creates recessive disruption in *h*. The size of the dots represents egg sample size from a single female. The rate estimates and corresponding estimates of the standard error of the mean (SEM) are displayed, obtained from pooling offspring from all crosses of the same type together (*n* = 1144 for the progeny of drive females, *n* = 464 for the progeny of drive males, and *n* = 655 for the progeny of *w*^*1118*^ individuals). An alternative statistical analysis that accounts for potential batch effects was also performed but yielded overall similar rates with only slightly increased error estimates (Supplementary Methods, Source Data: Data Sets 1−3).

To confirm the mechanism of action of our TARE drive, we crossed drive/wild-type heterozygotes with one copy of the Cas9 allele with *w*^*1118*^ flies, and also crossed male and female *w*^*1118*^ flies together. Individual flies were then allowed to lay eggs for up to three 20-h intervals. Eggs were counted at the end of these intervals, and subsequently, pupae were also counted in addition to phenotyping of eclosed adults. Counts of vials adversely affected by fungal growth were discarded to reduce variability in the egg-to-pupae survival rate, though later growing fungus did result in a higher death rate for pupae in some remaining vials when vial fly density was low. We found that female drive heterozygotes had 49.2% egg-to-pupae survival (Fig. 5, Supplementary Data Set 1) and male drive heterozygotes had 79.5% survival (Fig. 5, Supplementary Data Set 2), compared to 82.9% for *w*^*1118*^ individuals without the drive and Cas9 (Fig. 5, Supplementary Data Set 3) and 80.5% for individuals homozygous for the Cas9 allele (Supplementary Data Set 3). Thus, while progeny of male drive heterozygotes had an egg-to-pupae survival rate that was comparable to *w*^*1118*^ individuals, the egg-to-pupae survival rate of progeny from female drive heterozygotes was only about 60% that of *w*^*1118*^ individuals, which was significantly lower (*p* < 0.001, Fisher’s exact test) and consistent with our results for drive inheritance in a model in which early embryo Cas9 activity results in the death of most flies not inheriting the drive allele (Supplementary Data Set 1).

### TARE drive cage study

To study the performance of the TARE drive in large cage populations, we first crossed drive homozygous males to females homozygous for the Cas9 allele. TARE drive/Cas9 carriers were crossed together for several generations, selecting in particular individuals with the brightest dsRed and EGFP phenotype. When individuals were confirmed to be homozygous for both the drive and Cas9 alleles, they were crossed to Cas9 homozygotes with no drive, generating individuals that were drive/wild-type heterozygotes at the drive locus, but still possessing two copies of the Cas9 allele. These were crossed to *w*^*1118*^ males. The resulting dsRed inheritance was 91.1%, which was only slightly higher than for drive/wild-type heterozygotes (*p* = 0.027, Fisher’s exact test), most likely because of the increased maternal deposition of Cas9 due to a second copy in the genome. However, this difference was small, implying that the split drive in a genetic background homozygous for Cas9 would likely have similar performance to a complete TARE drive (where Cas9 would be included in the drive construct) in a wild-type background.

**Fig. 6:**
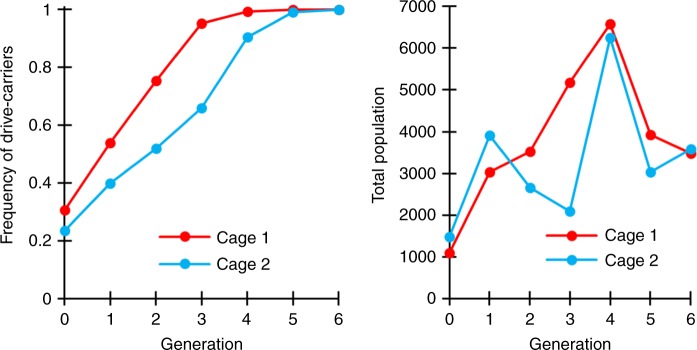
Cage frequency trajectories and population sizes. In large cages, individuals homozygous for the drive allele and the Cas9 allele were mixed with individuals homozygous only for the Cas9 allele. Cage populations were maintained for several discrete generations, and all individuals from each generation were phenotyped for dsRed, indicating that they carried one or two copies of the drive allele.

Flies homozygous for both the drive allele and the Cas9 allele were then expanded and allowed to lay eggs in bottles for one day. Flies homozygous for only the Cas9 allele were allowed to similarly lay eggs for 1 day in another set of bottles. Flies were then removed, and the bottles were placed in varying proportions in two population cages. Emerging adults were all homozygous and were considered to be “generation zero”. These were followed for several generations, with each generation (including generation zero) phenotyped for dsRed (Fig. 6, Supplementary Data Set 4). The cages were terminated when 100% of the population were found to be drive carriers since at this point, it is likely that all wild-type alleles were converted to disrupted alleles, making the remaining behavior of the drive predictable.

The evolution of r1 resistance alleles (which preserve the function of *h*) could pose a critical problem for a TARE drive. Our experimental design allows us to determine an upper bound for the rate at which such r1 resistance alleles were generated during the spread of the drive by phenotyping the population for dsRed at the point when the drive has reached all individuals in the cage. At this stage, all wild-type alleles are disrupted, but the drive has not fixed. Our model predicts that approximately 17% of individuals should then still be heterozygous for a drive and a disrupted *h* allele at this point (Fig. 2b). In the absence of large fitness costs to the drive, drive and r1 alleles should behave similarly at this stage, since both provide rescue. Thus, 17% of r1 alleles in our cages would be predicted to be paired with a disrupted *h* allele, and these individuals would not have dsRed phenotype. Since all individuals in both cages had dsRed phenotype at the end of our cage study, this indicates that either no r1 alleles formed in both cages, or that they were present at very low frequencies below the limit of detection. Assuming a model of binomial sampling, an r1 frequency of 0.23% in the population would have allowed us a 95% chance of detecting at least one r1 allele in our cages (see Supplementary Data Set 4 for details), so the actual frequency that such alleles are formed is likely below this level.

Interestingly, both cages seemed to outperform the predictions from a deterministic model assuming perfect drive efficiency and no fitness costs. A caveat to this is that we used a split-drive configuration, and thus, we had no power to detect fitness costs associated with the expression of the large Cas9 protein, which may represent a substantial fraction of a complete TARE drive’s fitness costs. In addition, drive individuals initially added to the cage may have been healthier, and maternal effects could have passed on this advantage with drive alleles for a few generations before it dissipated, which could have allowed the drive allele to somewhat outperform the predictions of the theoretical model. Finally, the recoded *h* allele itself may be advantageous in a cage setting compared to a wild-type allele. None of these factors would likely allow a TARE drive to have a fitness greater than wild-type individuals in a natural setting, particularly if it was carrying a payload gene.

## Discussion

In this study, we demonstrated the design of a same-site TARE system that was capable of quickly spreading through two experimental cage populations without apparent evolution of resistance. Although we tested our system only in *D. melanogaster*, it should be straightforward to transfer such a system to other organisms, such as mosquitoes, as long as one can find a suitable haplosufficient target gene where disrupted alleles are recessive lethal. A TARE drive would likely be successful even if lethality or haplosufficiency is incomplete, allowing for an even broader class of potential target genes. In the latter case, the introduction threshold frequency would be slightly increased even without fitness costs, which may be desirable for better confinement (see a study on the similar ClvR system for a detailed analysis of haploinsufficiency^38^—TARE would be expected to behave similarly).

While we used the promoter of the highly conserved germline-expressed gene *nanos* for Cas9 expression in our construct, a TARE drive should work with a variety of promoters that are active in gametes or their precursor cells, though maternal activity would still be highly desirable for rapid spread. This makes such a system considerably more flexible than CRISPR homing-type drives, where embryo activity can be problematic due to its propensity for forming undesirable resistance alleles^15,16^. In contrast, such embryo activity actually helps the drive spread faster for a TARE system. As a result, TARE systems are far less prone to the formation of resistance alleles than homing-type drives, particularly for population modification drives. We used two gRNAs in our drive construct, but with the same tRNA system, four or more gRNAs could easily be expressed, further reducing the potential for r1 allele formation. Such gRNA multiplexing would presumably have no negative effects on drive efficiency, unlike for homing drives^16^.

One caveat is that TARE systems, similar to homing drives with recoded regions, may still be vulnerable to the formation of r1 resistance alleles by undesired homology-directed repair (either after drive cleavage or by normal chromosomal recombination) that includes the recoded target region (though this would not be an issue if the payload gene is also included). To reduce the rate at which this may take place, one should take care to minimize regions of homology, as we performed in this study by selecting targets a large distance from the drive insertion site. If the native 3′UTR sequence is used in the recoded region, then a smaller 3′UTR would be preferred, as well as a larger distance between the target site and 3′UTR. Additional measures could involve the use of a substitute or recoded 3′UTR. Rearrangement of recoded regions could further minimize undesired homology-directed repair, as proposed in a study on *Medea*^27^.

**Fig. 7:**
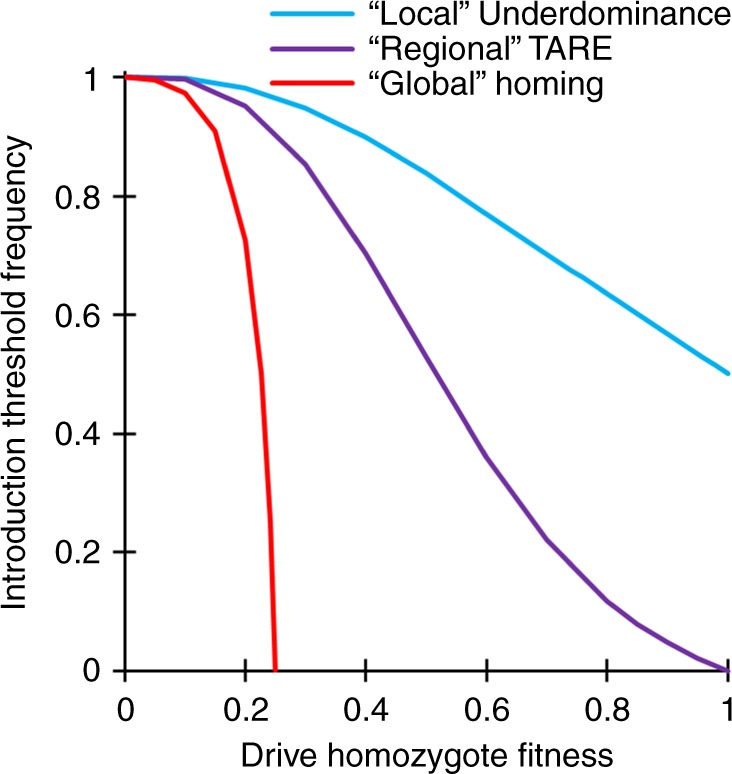
Introduction threshold comparison. The chart shows the introduction threshold frequencies for drive homozygotes as a function of the drive fitness cost in homozygotes using a deterministic model of a single panmictic population. These thresholds represent unstable equilibria above which the drive increases in frequency and below which the drive is removed. All drives are idealized, and the underdominance system uses a single locus with heterozygote disadvantage^44,51^.

A TARE drive shows threshold-dependent dynamics, usually with a low threshold if the drive has a fitness cost or the target gene is not completely haplosufficient. Thus, it would likely spread rapidly in the release region, but fail to establish in other populations from rare long-distance migrants. This is in contrast to “global” drives (such as homing drives), which could spread successfully after long-distance dispersal of even just a few individuals^35^. Nevertheless, due to their comparatively low introduction thresholds (if fitness costs are low), TARE systems are still expected to be more invasive than proposed underdominance systems^43–45,47,48^, which are commonly referred to as “local” drives. We therefore propose to classify TARE drives in a separate category termed “regional” systems (which would also include *Medea*^27^ and other similar toxin-antidote systems^38,50^) that lies between global and local drives. More specifically, we define a drive to be “regional” if it has an introduction threshold of zero only in an idealized model of a perfectly efficient drive without any additional fitness costs, while any degree of imperfection (as would seem hard to avoid for any such system in practice) would give rise to a nonzero introduction threshold (Fig. 7). Such a regional drive would then have the ability to spread within connected regions and between populations linked by moderate migration, but not to invade another population from a single seeding of a low number of migrants or from a very small continuous migration rate. This is in contrast to “global” drives that have a zero threshold even in the presence of moderate fitness costs, and “local” drives that still have a moderate threshold without any fitness costs (Fig. 7). Of course, as drive fitness costs increase, drives classified as regional and global could become more confined. However, if these fitness costs are caused by a payload, the drive may become less confined if it loses the payload (potentially resulting in the spread of the drive to a larger area than the payload).

If additional confinement beyond a standard “regional” TARE system is desired, then a local TARE-based underdominance system consisting of two TARE alleles, each targeting the gene the other provides rescue for, would presumably be relatively straightforward to design and engineer. This is particularly the case in comparison to other underdominance systems that rely on complex RNAi or chromosomal translocations. Another possibility for creating a local drive would be a TARE-like system targeting a gene with a high degree of haploinsufficiency.

TARE systems do have some limitations, such as the extended period it can take them to go from high frequency to ultimate fixation (or equilibrium). Indeed, a TARE drive will not be predicted to fixate if it has any additional fitness cost, though one copy will still be present in all individuals if r1 resistance alleles can be avoided. Thus, TARE drive should be particularly suitable for cases in which a single drive allele in all individuals is sufficient to provide the desired population modification effect. By using an X-linked target gene, fixation would be possible even with fitness costs, but at the cost of slower drive spread^50^. Another limitation of TARE drives is that they cannot be used for population suppression, though other “TA” systems could possibly do so, at the cost of greater construction difficulty^50^. This limitation could potentially be overcome by using a “tethered” homing-type suppression drive^37^, either with a TARE system or a TARE-based two-locus underdominance system if a higher invasion threshold is desired.

TARE systems can be “same-site” or ClvR-type^38^ drives at a “distant-site”. In the former, as demonstrated here, the drive allele is located at the target site gene, while in the latter the drive allele is located at a different locus in the genome, usually unlinked from the target gene. For the relatively high germline and embryo cut rates demonstrated thus far, both systems have similar population dynamics^50^. However, same-site systems could require an antidote element of reduced size, since natural promoter elements are used, enabling easier engineering. This has the advantage of ensuring that a different genomic location and/or an incomplete promoter will not affect expression of the recoded target gene compared to the wild-type gene, enhancing the chance of successful rescue and likely reducing fitness costs. Distant site systems, on the other hand, may be advantageous if the aim is to disrupt a gene other than the target gene, since the gene could be reliably disrupted by the presence of the drive allele without the need to target it with additional gRNAs. They could also potentially be used with smaller target genes if same-site systems would struggle to avoid undesired homology-directed repair of rescue elements. However, if homology-directed repair is possible between a distant site drive and the target site, this potential advantage may be mitigated.

Our study shows that TARE systems are promising candidates for regionally confined population modification drives and do not suffer from the high resistance rates typically observed in homing-type drives. With their great flexibility in choosing promoters and target sites, such drives could potentially be developed in a wide variety of organisms with reduced development time compared to other drive mechanisms.

## Methods

### Simulations

Deterministic, discrete-generation simulations were initialized by seeding a population of wild-type individuals with drive/wild-type heterozygous individuals at a specified introduction frequency. Each female individual selects a mate randomly in each generation. The probability of a male to be chosen is proportional to its fitness value. Females then generate offspring at a rate proportional to their fitness value. Fitness costs per drive allele are assumed to be codominant and multiplicative. In this model, fitness values represent fecundity for females and mating success for males relative to wild-type individuals. For the idealized homing drive, the wild-type allele is converted to a drive allele in the germline of drive heterozygotes at 100% efficiency. For the idealized TARE system, the wild-type target gene is disrupted in the germline of drive heterozygotes and in the early embryo of any individual if their mother has a drive allele at 100% efficiency. Individuals with two disrupted alleles are nonviable. For *Medea*, offspring are nonviable if their female parent had a drive allele and they did not inherit a drive allele from either parent. Fitness costs are assumed to be multiplicative (they are specified for homozygotes, while heterozygotes have a fitness equal to the square root of this value). For the underdominance drive, heterozygotes have their fitness further multiplied by a factor of 0.26, inspired by a previous experimental demonstration of such a system^44,51^. At the end of a generation cycle, offspring genotype frequencies are normalized to generate the final allele frequencies. This model was used to generate the data in Figs. 2 and  7.

### Plasmid construction

The starting plasmids pCFD3^52^ (Addgene plasmid #49410) and pCFD5^53^ (Addgene plasmid #73914) were kindly supplied by Simon Bullock, and starting plasmid IHDyi2 was constructed in our previous study^15^. All plasmids were digested with restriction enzymes from New England Biolabs (HF versions, when possible). Polymerase chain reaction (PCR) was conducted with Q5 Hot Start DNA Polymerase (New England Biolabs) with DNA oligos and gBlocks from Integrated DNA Technologies. Gibson assembly of plasmids was conducted with Assembly Master Mix (New England Biolabs), and plasmids were transformed into JM109 competent cells (Zymo Research). Plasmids used for injection into eggs were purified using the ZymoPure Midiprep kit (Zymo Research). Cas9 gRNA target sequences were identified using CRISPR Optimal Target Finder^54^. Tables of DNA fragments used for Gibson Assembly of each plasmid, PCR products with the oligonucleotide primer pair used, and plasmid restriction digests with the restriction enzymes are shown in the Supplementary Methods. ApE files of all plasmids and alleles are also included as supplementary material.

### Generation of transgenic lines

Lines were transformed at Rainbow Transgenic Flies by injecting the donor plasmid (EGDh2) into a *w*^*1118*^ line. Plasmid pHsp70-Cas9^55^ (provided by Melissa Harrison & Kate O’Connor-Giles & Jill Wildonger, Addgene plasmid #45945) was included as a source of Cas9 and plasmid EGDhg2t was included as a source of gRNA in the injection. Injection concentrations of donor, Cas9, and gRNA plasmids were 314, 313, and 63 ng/µL, respectively in 10 mM Tris-HCl, 100 µM Ethylenediaminetetraacetic acid (EDTA), pH 8.5 solution. To obtain homozygous lines, injected individuals were first crossed with *w*^*1118*^ flies. The progeny with dsRed fluorescent protein in the eyes, which usually indicated successful insertion of the drive, were then crossed with each other for several generations, with preference to flies with slightly brighter eyes, which usually indicated that the individual was homozygous for the drive. The stock was considered homozygous after sequencing confirmation. The split-CRISPR line with Cas9 expressed by the *nanos* promoter was generated as part of a previous study^17^.

### Fly rearing and phenotyping

All flies were reared at 25 °C with a 14/10 h day/night cycle. Bloomington Standard medium was provided as food every 2 weeks. For phenotyping, flies were anesthetized with CO_2_ and examined with a stereo dissecting microscope. Red fluorescent eye phenotypes were scored using the NIGHTSEA system (SFA-GR). The different phenotypes and genotypes of our drive system are summarized in Supplementary Data Sets 1−2.

For the cage study, enclosures of internal dimensions 30 × 30 × 30 cm (Bugdorm, BD43030D) were used to house flies. At the start of an experiment, drive flies and split-Cas9 flies were crossed as above until found to be homozygous at both sites by higher red and green fluorescent brightness and confirmed by subsequent crosses with wild-type individuals. These, together with split-Cas9 flies of the same age, were separately allowed to lay eggs in eight food bottles for 1 day. Bottles were then placed in cages at the desired starting ratios between drive and nondrive flies. Eleven days later, bottles were replaced in the cage with fresh food, leaving adult flies in the cages. One day later, bottles were removed from the cages, the flies were frozen for later phenotyping, and bottles were returned to the cage. This 12-day cycle was repeated for each subsequent generation.

All experiments involving live gene drive flies were carried out using Arthropod Containment Level 2 protocols at the Sarkaria Arthropod Research Laboratory at Cornell University, a quarantine facility constructed to comply with containment standards developed by USDA APHIS. Additional safety protocols regarding insect handling approved by the Institutional Biosafety Committee at Cornell University were strictly obeyed throughout the study, further minimizing the risk of accidental release of transgenic flies. All drive flies also utilized our split-Cas9 system^17^, which should prevent the spread of the drive in the case of an accidental escape.

### Genotyping

To obtain the DNA sequences of gRNA target sites, flies were frozen and then homogenized in 30 µL of 10 mM Tris-HCl pH 8, 1 mM EDTA, 25 mM NaCl, and 200 µg/mL recombinant proteinase K (Thermo Scientific). The mixture was incubated at 37 °C for 30 min and then 95 °C for 5 min. The solution was used as the template for PCR to amplify the gRNA target site. DNA was purified by gel extraction and Sanger sequenced. Sequences were analyzed using the ApE software (2.0.60), available at: http://biologylabs.utah.edu/jorgensen/wayned/ape.

### Reporting summary

Further information on research design is available in the Nature Research Reporting Summary linked to this article.

## Supplementary information

Supplementary Information

Reporting Summary

## Data Availability

The data sets involved in the current study are available in the manuscript and supplementary material. Any other relevant data are available from the authors upon reasonable request. Source data are available in the Source Data file.

## Source Data

Source Data
