# Supplementary material for: A toxin-antidote CRISPR gene drive system for regional population modification
Source: Nat Commun. 2020 Feb 27;11:1082. doi: 10.1038/s41467-020-14960-3 (PMC7046741; doi:10.1038/s41467-020-14960-3)
Supplement: Supplementary file 1 — Supplementary Information [file 41467_2020_14960_MOESM1_ESM.pdf]

Supplementary Information

A toxin-antidote CRISPR gene drive system for regional population modification

Champer *et al.*

Contents:

Supplementary Methods
Supplementary Figures 1-3
Supplementary Table 1

Supplementary Methods

The following tables show the DNA fragments used for Gibson Assembly of each plasmid and the sequences of DNA oligos. PCR products are shown with the oligonucleotide primer pair used, and plasmid digests are shown with the restriction enzymes used.

Plasmid for assembly of the gRNAs used in the drive:

**EGDhg2**	*Template*	*Oligo/Enzyme 1*	*Oligo/Enzyme 2*
PCR Product	pCFD3	CFD5h_1_F	CFD_1_R
PCR Product	pCFD5	CFDh_12_F	CFDh_12_R
PCR Product	pCFD5	CFD_2_F	CFD5h_2_R

Plasmid for expression of the gRNAs used to transform the drive into the genome:

**EGDhg2t**	*Template*	*Oligo/Enzyme 1*	*Oligo/Enzyme 2*
PCR Product	pCFD3	CFD5ht_1_F	CFD_1_R
PCR Product	pCFD5	CFDht_12_F	CFDht_12_R
PCR Product	pCFD5	CFD_2_F	CFD5ht_2_R

Intermediate for construction of the drive plasmid:

**EGDh2i1**	*Template*	*Oligo/Enzyme 1*	*Oligo/Enzyme 2*
PCR Product	Genomic DNA	hLeft_F	hLeft_R
PCR Product	gBlock ghr	hcode_F	hcode_R
PCR Product	Genomic DNA	h3utr_F	h3utr_R
Plasmid Digest	IHDyi1*	AvrII	XmaI

*a plasmid backbone derived from IHDyi1, with the BsiWI site replaced by an XmaI site

Drive plasmid designed to incorporate the drive into in the genome:

**EGDh2**	*Template*	*Oligo/Enzyme 1*	*Oligo/Enzyme 2*
PCR Product	EGDhg2	U6_h_F	U6_h_R
PCR Product	Genomic DNA	hRight_F	hRight_R
Plasmid Digest	EGDh2i1	AgeI	XhoI

Construction primers

```
CFD_1_R: GGCTATGCGTTGTTTGTTCTGC
CFD_2_F: AACAGTAGGCAGAACAAACAACGC
CFD5h_1_F: GTGCAGCTGCTGCTGGGGCAGGCTCGTTTTAGAGCTAGAAATAGCAAGTTAAA
CFD5h_2_R: AAAACACTGTGTGAACGAGGTTAGCTGCATCGGCCGGGAATCGAAC
CFD5ht_1_F: GTGCAACGGTCACTTTTGAGCGGCGGTTTTAGAGCTAGAAATAGCAAGTTAAA
CFD5ht_2_R: AAAACCCGTTGTGCCGGCCCAGCTCTGCATCGGCCGGGAATCGAAC
```

```
CFDh_12_F: ATGCAGCTAACCTCGTTCACACAGTGTTTTAGAGCTAGAAATAGCAAGTTAAA
CFDh_12_R: AAAACGAGCCTGCCCCAGCAGCAGCTGCACCAGCCGGGAATCGAAC
CFDht_12_F: ATGCAGAGCTGGGCCGGCACAACGGGTTTTAGAGCTAGAAATAGCAAGTTAAA
CFDht_12_R: AAAACCGCCGCTCAAAAGTGACCGTTGCACCAGCCGGGAATCGAAC
h3utr_F: GTAAAGGGTGTCTGCATATGCATATCA
h3utr_R: AATTGAATTAGTCTCTAATTGAATTAGATCCGAGCTCACCCAGGAAAAGATACCCCAAC
hcode_F: CGTTCTGGGCACCGCCGTGGTCCCCGCTCAA
hcode_R: ATATGATATGCATATGCAGACACCCTTTACCAC
hLeft_F: ATTAACCAATTCTGAACATTATCGCCTAGGGTACCGAAGCAGCAACAACACCAACACCAC
hLeft_R: TGAGCGGGGACCACGGCGGTGCCCAGAACGTT
hRight_F: TTAATGCGTATGCATCGCTCAAAAGTGACCGTCG
hRight_R: TCGCCCTTGAACTTGATTGACGGAAGAGCCTCGAGTCGATTAGTCACGGCTTTTGC
U6_h_F: GGTTTGTCCAAACTCATCAATGTATCTTAACGCGTTTTTTTGCTCACCTGTGATTGC
U6_h_R: GGTCACTTTTGAGCGATGCATACGCATTAAGCGAACA
```

Sequencing primers

```
h3utr_S_F: AAGGACCTTCATCAGACGCAC
h3utr_S_R: GTTTGGCGATGTCCTGGTAGA
hCut_S_F: CCAAATTGGAAAAGGCCGACA
hCut_S_R: AACATGGGTTGCTGTTGTGC
hLeft_S_F: TCAGATTTGCTGCCAAGTGAAA
hLeft_S_R: CCAGAACGTTGGTCATGTTGG
hRec_S_F: TCATGCTCAAATGTTGCCGAG
hRec_S_R: AATGACTTGCATTCCGTTCGG
hRight_S_F: CGTGCAAAAGCCGTGACTAAT
hRight_S_R: TAGTAAATGCCACCAACGCGA
pCFD5_S_R: ACGTCAACGGAAAACCATTGTCTA
```

These sequencing primers were all used to confirm plasmid sequences. For genotyping PCR, the primers hCut_S_F and hCut_S_R were used to determine sequences of cut sites as described in the methods.

gBlock

```
ghr:
GCCGTGGTCCCCGCTCAACTGAAAGAAACCCCCCTGAAGAGCGATCGCCGCAGCAATAAACCGATTATGGAAAAGCG
TCGCCGCGCTCGCATCAATAATTGCCTGAACGAGCTGAAAACCTTGATCTTGGACGCTACGAAGAAGGATCCCGCCC
GTCATAGCAAGCTGGAGAAAGCTGATATCTTGGAAAAAAACCGTGAAACACTTGCAAGAATTGCAACGTCAACAAGCC
GCTATGCAACAAGCTGCTGACCCGAAAATCGTCAATAAGTTTAAAGCTGGCTTTGCTGATTGCGTCAATGAAGTGTC
CCGTTTCCCGGGAATTGAACCGGCTCAACGCCGCCGTTTGCTGCAACATTTGTCCAATTGTATTAACGGAGTGAAAA
CCGAATTGCATCAACAACAACGTCAACAACAGCAACAAAGCATTCATGCTCAAATGTTGCCGAGCCCCCCGTCCAGC
CCCGAACAAGACTCCCAACAAGGCGCCGCCGCCCCGTATCTGTTCGGCATTCAACAAACCGCTTCCGGCTATTTCTT
GCCGAACGGAATGCAAGTCATTCCGACGAAATTGCCGAATGGCTCCATCGCTCTGGTCCTGCCGCAATCCCTGCCGC
AACAGCAACAGCAACAGCTGCTCCAACATCAACAGCAACAGCAACAGTTGGCCGTGGCTGCCGCTGCGGCCGCTGCC
GCTGCCGCGCAGCAACAGCCGATGCTGGTGTCCATGCCGCAACGCACCGCTTCCACGGGCAGCGCTTCCAGCCATAG
CAGCGCTGGCTATGAAAGCGCCCCGGGCTCCTCCTCCTCCTGCTCCTATGCTCCCCCGAGCCCCGCTAATAGCTCCT
```

ATGAACCGATGGATATTAAACCCAGCGTGATTCAACGTGTCCCCATGGAACAACAACCGTTGAGCTTGGTCATTAAG
AAACAGATTAAGGAAGAAGAACAACCGTGGCGCCCGTGGTAAAGGGTGTCTGCATATGCATATC

Drive parameter calculations

Drive parameters were estimated by counting the number of flies with specific phenotypes among the progeny from crosses of D/+ heterozygotes with wild-type individuals. To calculate rates from these counts, we employed two different approaches. In the first approach, we simply pooled all offspring from the same type of cross together and then calculated rates from the combined overall counts of progeny with the listed phenotypes according to following equations:

Drive inheritance rate = $\frac{\text{\#dsRed flies}}{\text{\#total flies}}$

Egg-to-pupae survival rate = $\frac{\text{\#pupae}}{\text{\#eggs}}$

Egg-to-pupae survival rate relative to wild-type = $\frac{\text{egg-to-pupae survival rate}}{\text{egg-to-pupae survival rate of wild-type}}$

Expected egg-to-pupae survival rate relative to wild-type = $\frac{0.5}{\text{drive inheritance}}$
(based off the expectation of an essential but haplosufficient target gene)

The resulting rates are shown in Figures 4 and 5, together with standard errors of the mean (SEM) obtained under a model of binomial sampling. All specific calculations are provided in the Supplementary Data Sets 1-3.

A potential issue of this pooling approach is that batch effects (groups of offspring were raised in separate vials with different parents) could distort the rate and error estimates. In our second approach we sought to account for such effects by using a generalized linear mixed-effects model with a binomial distribution (fit by maximum likelihood, Adaptive Gauss-Hermite Quadrature, nAGQ = 25). Such a model allows for variance between batches, resulting in potentially different rate estimates and increased error estimates. Offspring from a single vial were considered as a separate batch, even if they had the same parents with offspring from other vials. This analysis was performed using the R statistical computing environment (3.6.1) with the packages lme4 (1.1-21, https://cran.r-project.org/web/packages/lme4/index.html) and emmeans (1.4.2, https://cran.r-project.org/web/packages/emmeans/index.html). The specific R script we used for this analysis is available on Github (https://github.com/MesserLab/Binomial-Analysis). The resulting rate estimates and errors from this alternative analysis were similar to the pooled analysis and are provided in the Supplementary Data Sets 1-3.

```
original      ATGGTTACCGGCGTAACAGCAGCCAACATGACCAACGTTCTGGGCACCGCCGTTGTGCCG  60   first recoded codon
recoded       ATGGTTACCGGCGTAACAGCAGCCAACATGACCAACGTTCTGGGCACCGCCGTGGTCCCC  60
              ***************************************************** ** **
                                                          intron in original sequence
original      GCCCAGCTCAAGGAGACGCCGCTCAAAAGTGACCGTCGGTCGAACAAGCCCATCATGGAG  120
recoded       GCTCAACTGAAAGAAACCCCCCTGAAGAGCGATCGCCGCAGCAATAAACCGATTATGGAA  120
              ** ** ** ** ** ** ** ** ** ** ** ** **    ** ** ** ** *****

original      AAACGCCGACGTGCCCGTATTAACAACTGTCTCAATGAACTCAAGACTCTGATTCTGGAT  180
recoded       AAGCGTCGCCGCGCTCGCATCAATAATTGCCTGAACGAGCTGAAAACCTTGATCTTGGAC  180
              ** ** ** ** ** ** ** ** ** ** ** ** ** ** ** **  ****  ****
                             intron in original sequence
original      GCCACCAAAAAAGACCCGGCTCGCCACTCCAAATTGGAAAAGGCCGACATTCTGGAGAAG  240
recoded       GCTACGAAGAAGGATCCCGCCCGTCATAGCAAGCTGGAGAAAGCTGATATCTTGGAAAAA  240
              ** ** ** ** ** ** ** ** **   ***  **** ** ** ** **  **** **

original      ACAGTAAAGCATCTGCAGGAGCTGCAGCGCCAGCAGGCAGCCATGCAGCAGGCCGCCGAT  300
recoded       ACCGTGAAACACTTGCAAGAATTGCAACGTCAACAAGCCGCTATGCAACAAGCTGCTGAC  300
              ** ** ** **  **** **  **** ** ** ** ** ** ***** ** ** ** **

original      CCCAAGATTGTGAACAAATTCAAGGCCGGATTCGCCGACTGTGTGAACGAGGTTAGCCGC  360
recoded       CCGAAAATCGTCAATAAGTTTAAAGCTGGCTTTGCTGATTGCGTCAATGAAGTGTCCCGT  360
              ** ** ** ** ** ** ** ** ** ** ** ** ** ** ** ** ** **   ***

original      TTTCCCGGCATCGAGCCCGCCCAGCGTCGTCGCCTGCTACAGCACCTGAGCAACTGCATC  420
recoded       TTCCCGGGAATTGAACCGGCTCAACGCCGCCGTTTGCTGCAACATTTGTCCAATTGTATT  420
              ** ** ** ** ** ** ** ** ** ** **  **** ** **  **  *** ** **

original      AATGGCGTTAAGACAGAGCTGCACCAGCAGCAGCGCCAGCAGCAACAGCAGTCCATCCAC  480
recoded       AACGGAGTGAAAACCGAATTGCATCAACAACAACGTCAACAACAGCAACAAAGCATTCAT  480
              ** ** ** ** ** **  **** ** ** ** ** ** ** ** ** **   *** **

original      GCCCAGATGCTGCCCTCGCCGCCCAGCTCGCCGGAGCAGGATAGCCAGCAGGGAGCAGCG  540
recoded       GCTCAAATGTTGCCGAGCCCCCCGTCCAGCCCCGAACAAGACTCCCAACAAGGCGCCGCC  540
              ** ** *** ****    ** **   *   ** ** ** **   *** ** ** ** **

original      GCACCCTACCTCTTTGGTATCCAGCAGACGGCCAGCGGTTACTTTCTGCCCAATGGCATG  600
recoded       GCCCCGTATCTGTTCGGCATTCAACAAACCGCTTCCGGCTATTTCTTGCCGAACGGAATG  600
              ** ** ** ** ** ** ** ** ** ** **   *** ** **  **** ** ** ***

original      CAGGTGATCCCCACCAAGCTGCCCAACGGTAGCATTGCCCTCGTGTTGCCCCAGAGCCTG  660
recoded       CAAGTCATTCCGACGAAATTGCCGAATGGCTCCATCGCTCTGGTCCTGCCGCAATCCCTG  660
              ** ** ** ** ** **  **** ** **   *** ** ** **  **** **   ****

original      CCCCAGCAGCAGCAGCAACAGTTGCTGCAGCACCAACAGCAGCAGCAGCAACTCGCCGTC  720
recoded       CCGCAACAGCAACAGCAACAGCTGCTCCAACATCAACAGCAACAGCAACAGTTGGCCGTG  720
              ** ** ***** ********* **** ** ** ******** ***** **  * *****

original      GCAGCAGCAGCAGCGGCCGCAGCAGCAGCACAACAGCAACCCATGTTGGTCAGCATGCCC  780
recoded       GCTGCCGCTGCGGCCGCTGCCGCTGCCGCGCAGCAACAGCCGATGCTGGTGTCCATGCCG  780
              ** ** ** ** ** ** ** ** ** ** ** ** ** ** *** ****   ******

original      CAGCGTACAGCCAGCACCGGATCCGCCAGCTCGCACTCCTCCGCCGGATACGAGTCGGCG  840
recoded       CAACGCACCGCTTCCACGGGCAGCGCTTCCAGCCATAGCAGCGCTGGCTATGAAAGCGCC  840
              ** ** ** **   *** **   ***   *   **   *  *** ** ** **    **

original      CCCGGAAGCAGCAGCAGCTGTAGCTACGCCCCGCCCAGTCCGGCCAACTCTAGCTACGAG  900
recoded       CCGGGCTCCTCCTCCTCCTGCTCCTATGCTCCCCCGAGCCCCGCTAATAGCTCCTATGAA  900
              ** **   *  *  *  ***   *** ** ** ** ** ** ** **      *** **

original      CCCATGGACATCAAGCCATCGGTCATCCAGCGCGTGCCAATGGAGCAGCAGCCCCTGTCG  960
recoded       CCGATGGATATTAAACCCAGCGTGATTCAACGTGTCCCCATGGAACAACAACCGTTGAGC  960
              ** ***** ** ** **    ** ** ** ** ** ** ***** ** ** **  **

original      CTGGTGATCAAGAAGCAGATCAAGGAGGAGGAGCAGCCCTGGCGGCCCTGGTAG1014
recoded       TTGGTCATTAAGAAACAGATTAAGGAAGAAGAACAACCGTGGCGCCCGTGGTAA1014
               **** ** ***** ***** ***** ** ** ** ** ***** ** *****
```

Supplementary Figure 1. Recoded *h* sequence alignment. Alignment between the original *h* sequence and the recoded version is shown, with “*” indicating an identical nucleotide. The locations of the first recoded codon and the two introns are also shown.

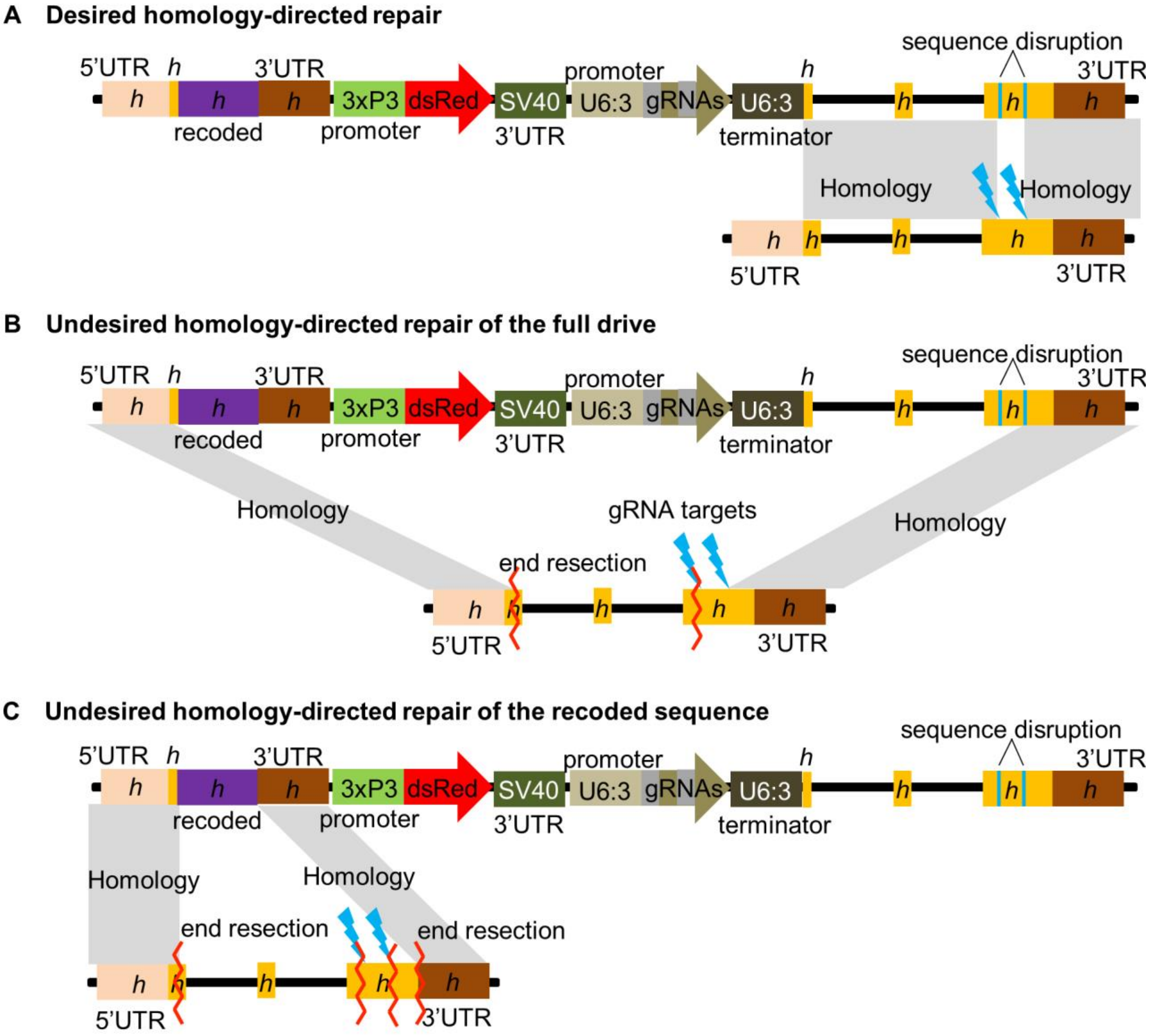

Supplementary Figure 2. Homology-directed repair possibilities in the TARE drive. (**A**) The homology between the drive construct and wild-type allele during "correct" homology-directed repair that results in copying the sequence disruptions in the drive allele. This form of homology-directed repair has the same effect as end-joining, with the added advantage that the repair is certain to disrupt the sequence (sequential end-joining events could potentially form a rare r1 resistance allele, depending on the sequence changes). (**B**) It is possible that homology-directed repair copies the entire drive allele. However, end resection must proceed for over ~1.4 kb on the left side of the cut sites, which may be rare, and this pathway would also be competing with the one in (A). If this happens at a small rate, overall drive dynamics are not likely to be affected. (**C**) It is also possible that homology-directed repair copies only the recoded sequence, immediately forming a functional r1 resistance allele. This would have a substantial negative effect on drive success. However, this event should be quite rare, since in addition to the end resection in (B), end resection would need to also proceed for over ~350 nucleotides to the right of the cut site, and it would then only be able to use the 3'UTR element near the recoded area as the homology template. It would also need to compete with the pathways in (A) and (B).

```
Wild Type:
Site 1: GATTCGCCGACT          GTGTGAACGAGGTTAGCCGCTTTCCC
Site 2: GTTGCCCCAGAG          CCTGCCCCAGCAGCAGCAGCAACAGT

"11" sequence
Site 1: GATTC-------          ------------------------CC
Site 2: GTTGCCCCAG--          ----------CAGCAGCAGCAACAGT

"13" sequence
Site 1: GATTCGCCGAC-          --------GAGGTTAGCCGCTTTCCC
Site 2: GTTGCCCCAGAG          --TGCCCCAGCAGCAGCAGCAACAGT
```

Supplementary Figure 3. Sequences of h cuts sites for the lines used in the cage experiment. Sequences of lines used in the experiment ("11" and "13") are displayed. Red indicates the sequences of gRNA target sites and orange indicates protospacer-adjacent motifs (PAMs). Purple dashed lines indicate deletions from the wild-type sequence.

Supplementary Table 1

Sequenced individual	Cut site 1	Cut site 2
Drive mother, dsRed offspring	mutated	wild-type
Drive mother, dsRed offspring	mutated	wild-type
Drive mother, dsRed offspring	wild-type	mutated
Drive mother, dsRed offspring	wild-type	mutated
Drive mother, dsRed offspring	wild-type	mosaic
Drive mother, dsRed offspring	wild-type	wild-type
Drive mother, wild-type offspring	mutated*	mutated*
Drive mother, wild-type offspring	mutated	wild-type
Drive mother, wild-type offspring	mutated	wild-type
Drive mother, wild-type offspring	mosaic	mosaic
Drive mother, wild-type offspring	mosaic	wild-type
Drive mother, wild-type offspring	wild-type	mutated
Drive father, wild-type offspring	mutated*	mutated*
Drive father, wild-type offspring	mutated	mutated
Drive father, wild-type offspring	mutated	mutated
Drive father, wild-type offspring	mutated	wild-type
Drive father, wild-type offspring	wild-type	mutated
Drive father, wild-type offspring	wild-type	wild-type

*Sequencing showed a deletion between both cut sites, indicating simultaneous cleavage.

Supplementary Table 1. Other *h* cut site sequence analysis. Due to the presence of multiple sequences in all individuals, *h* cut sites were distinguished by type (wild-type, mutated, or mosaic). dsRed offspring with a drive mother have a mutated cut sequence that is part of the drive allele. The table shows the type of the other allele. Similarly, the wild-type offspring all had a wild-type allele, and the sequence type shown is the type of the other allele. Mosaic sequences are formed by maternal Cas9 cleavage past the zygote stage, usually resulting in a high degree target sequence variation within an individual.
